# Residual Strain Evolution Induced by Crystallization Kinetics During Anti‐Solvent Spin Coating in Organic–Inorganic Hybrid Perovskite

**DOI:** 10.1002/advs.202205986

**Published:** 2023-04-25

**Authors:** Y. Sun, Q. Yao, W. Xing, H. Jiang, Y. Li, W. Xiong, W. Zhu, Y. Zheng

**Affiliations:** ^1^ Guangdong Provincial Key Laboratory of Magnetoelectric Physics and Devices School of Physics Sun Yat‐sen University Guangzhou 510275 China; ^2^ State Key Laboratory of Optoelectronic Materials and Technologies School of Physics Sun Yat‐sen University Guangzhou 510275 China; ^3^ Centre for Physical Mechanics and Biophysics School of Physics Sun Yat‐sen University Guangzhou 510275 China

**Keywords:** crystallization kinetics, organic–inorganic hybrid perovskite, polycrystalline thin film, residual strain, thin‐film mechanics

## Abstract

Organic–inorganic hybrid perovskite (OIHP) polycrystalline thin films are attractive due to their outstanding photoelectronic properties. The anti‐solvent spin coating method is the most widely used to synthesize these thin films, and the residual strain is inevitably originates and evolves during the process. However, this residual strain evolution induced by crystallization kinetics is still poorly understood. In this work, the in situ and ex situ synchrotron grazing‐incidence wide‐angle X‐ray scattering (GIWAXS) are utilized to characterize the evolution and distribution of the residual strain in the OIHP polycrystalline thin film during the anti‐solvent spin coating process. A mechanical model is established and the mechanism of the crystallization kinetics‐induced residual strain evolution process is discussed. This work reveals a comprehensive understanding of the residual strain evolution during the anti‐solvent spin coating process in the OIHP polycrystalline thin films and provides important guidelines for the residual strain‐related strain engineering, morphology control, and performance enhancement.

## Graphical abstract

The residual strain evolution during anti‐solvent spin coating of organic–inorganic hybrid perovskite thin‐film significantly affects its morphology and performance but is poorly understood. Based on the in situ and ex situ synchrotron grazing‐incidence wide‐angle X‐ray scattering, this work combines crystallization kinetics and thin‐film mechanics to establish a model and explore the mechanism underlying the residual strain distribution and evolution.

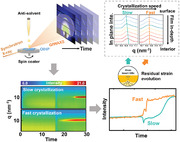

## 1 Introduction

Organic–inorganic hybrid perovskite (OIHP) polycrystalline thin films, with their outstanding electronic and photoelectronic properties, are hot candidate materials for various applications, such as solar cells, light emitting diodes, gas/light detectors and artificial synapses, etc.^[^ ^1^, ^2^, ^3^, ^4^, ^5^, ^6^ ^]^ The main fabrication methods of the OIHP polycrystalline thin films are solution methods,^[^ ^7^, ^8^ ^]^ including one‐step spin coating^[^ ^9^, ^10^ ^]^ and two‐step spin coating^[^ ^11^ ^]^ et al. In the one‐step spin coating methods, the anti‐solvent spin coating method is most widely used, owing to the high crystallinity in the OIHP polycrystalline thin film and the great coverage percentage on the substrate.^[^ ^9^, ^12^ ^]^ In this method, the OIHP undergoes a series of procedures driven by crystallization kinetics to completely transform into a polycrystalline thin film, which inevitably possesses the residual strain inside itself.^[^ ^13^, ^14^, ^15^ ^]^

The residual strain inside the OIHP polycrystalline thin film has advantages and disadvantages.^[^ ^16^ ^]^ On the one hand, a moderate compressive strain can increase its photoelectronic properties, stability^[^ ^17^, ^18^ ^]^ and flexibility.^[^ ^19^ ^]^ On the other hand, an excessive tensile or compressive strain could lead to the consequential wrinkle, crack or fracture of the film.^[^ ^20^, ^21^ ^]^ Currently, the residual strain can be intentionally regulated via the well‐designed epitaxial growth or atom substitution processes. Besides, it can also be naturally generated as a by‐product during the solidification and thermal treatment processes of the thin film. To date, many researchers have focused on the residual strain generated by epitaxial growth,^[^ ^22^, ^23^ ^]^ atom substitution,^[^ ^24^, ^25^ ^]^ and thermal treatment^[^ ^13^, ^26^ ^]^ in the OIHP polycrystalline thin films and discussed the related residual strain evolution processes and strain engineering strategies. However, the residual strain evolution inside the film during the crystallization process in the anti‐solvent spin coating method is still poorly understood. Practically, the crystallization process can generate a significant residual strain, and this residual strain could be the starting point for its evolution in the following thermal treatment. Furthermore, the residual strain evolution mechanism in the OIHP polycrystalline thin films fabricated by the anti‐solvent spin coating method could be different from that in the traditional chemical vapor deposition (CVD)^[^ ^27^ ^]^ or electroplating method.^[^ ^28^ ^]^ Current studies have shown that the solidification or crystallization of the OIHP polycrystalline thin films in the anti‐solvent spin coating method is based on the removal of the precursor solvent(s) and initiate crystallization of the perovskite film.^[^ ^29^, ^30^, ^31^ ^]^ As a result, rather than the traditional “bottom‐up” growth mode of the crystals from the precursor‐substrate interface to the surface in the CVD or electroplating method, the OIHP polycrystalline thin films via the anti‐solvent spin coating method tend to nucleate at the precursor‐gas interface and grow “downward” to the substrate (“top‐down” growth mode),^[^ ^32^, ^33^, ^34^, ^35^ ^]^ where the mechanism of the residual strain evolution should be completely different from that in the “bottom‐up” growth mode. In addition, the process of the anti‐solvent spin coating method is very fast and usually lasts only tens of seconds, and the fabricated thin film is about several hundreds of nanometers. The time‐sensitive feature and the thin film structure make it challengeable to comprehensively and thoroughly investigate the residual strain evolution mechanism in the anti‐solvent spin coating process. So, it is necessary to employ experimental characterization techniques with high spatial and temporal resolutions to facilitate the related researches.

In this work, we utilized the ex situ and in situ synchrotron grazing‐incidence wide‐angle X‐ray scattering (GIWAXS)^[^ ^36^, ^37^, ^38^, ^39^, ^40^, ^41^, ^42^, ^43^ ^]^ to characterize the residual strain evolution and distribution during and after the anti‐solvent spin coating process. The ex situ GIWAXS results show that the residual strain exhibits different distributions inside the OIHP polycrystalline thin films with different fabrication conditions. The in situ GIWAXS results collected during the anti‐solvent spin coating process show that the crystallization speed plays an important role in affecting the residual strain and film morphology. A mechanical model is established based on the experimental results to explore the influence of crystallization speed on the residual strain evolution and the film morphology. Furthermore, based on crystallization kinetics, the mechanism of the residual strain evolution during the anti‐solvent spin coating process is discussed. This work reveals a deeper and more comprehensive understanding of the residual strain evolution induced by crystallization kinetics during the anti‐solvent spin coating process of the OIHP polycrystalline thin films and provides important guidelines for the residual strain‐related strain engineering, morphology control and photo‐electronic performance improvement of the thin films.

## 2 Results

### 2.1 Residual Strain Distribution and Evolution in the OIHP Polycrystalline Thin Film

To study the residual strain distribution and evolution during the anti‐solvent spin coating process, the CH_3_NH_3_PbBr_3_ (MAPbBr_3_) polycrystalline thin film as the typical material was selected in this work. The MAPbBr_3_ film can fully crystallize during the anti‐solvent spin coating process and maintain the same morphology and optoelectronic properties without any further annealing process.^[^ ^9^ ^]^ This feature can remove the influence of the thermal treatment on the residual stain and ensure that the difference in the residual strain only results from the variety in the crystallization process.

**Figure 1:**
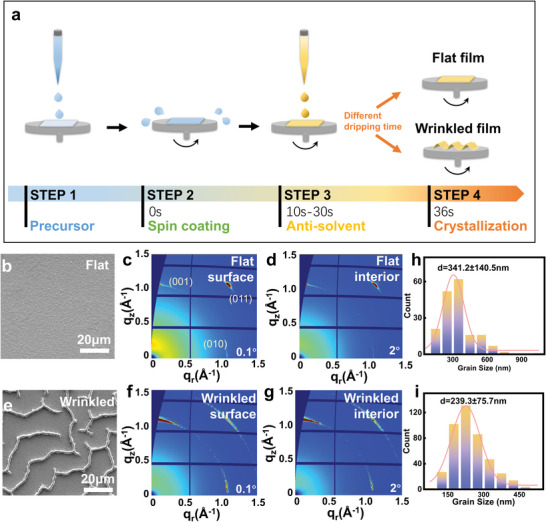
Process flow of the anti‐solvent spin coating of the MAPbBr_3_ polycrystalline thin film and its characterization results. a) Schematic of the anti‐solvent spin coating process for the flat or wrinkled MAPbBr_3_ polycrystalline thin film. b) Scanning electron microscope (SEM) image of the flat film. c,d) 2D GIWAXS patterns of the flat film with the incident angles of c) 0.1° and d) 2°, reflecting the crystal structure of the surface and interior of the thin film, respectively. e) SEM image of the wrinkled film. f,g) 2D GIWAXS patterns of the wrinkled film with the incident angles of f) 0.1°and g) 2°, reflecting the crystal structure of the surface and interior of the thin film, respectively. h,i) Grain size distributions of the h) flat and i) wrinkled thin films.

The main steps of the anti‐solvent spin coating method to fabricate the MAPbBr_3_ polycrystalline thin film is illustrated in **Figure** **1**a. Interestingly, with the different dripping times of the anti‐solvent, the film would come up with either a flat or wrinkled morphology, shown in Figure 1b,e, respectively. Based on the comparison between the X‐ray diffraction patterns of the wrinkled and flat thin films in Figure S1, Supporting Information, there's no residual PbBr_2_ or other secondary product in the final wrinkled MAPbBr_3_ thin film.

The ex situ synchrotron GIWAXS was used to examine the difference in phase or crystal structure of the films with different morphologies. Two incident angles of 0.1 ^°^ and 2 ^°^ were chosen to ascertain the crystal structures of the surface and interior of the thin film, respectively. The corresponding 2D GIWAXS patterns are shown in Figure 1c,d (flat film) and f,g (wrinkled film).^[^ ^44^, ^45^ ^]^ These 2D GIWAXS patterns show that both the flat film and wrinkled film exhibit the same MAPbBr_3_ cubic structure with a slight difference in the crystal orientation uniformity.^[^ ^46^, ^47^ ^]^ The average grain size of the flat film is larger than that of the wrinkled film, as shown in Figure 1h,i. The steady‐state photoluminescence (PL) spectra (Figure S2a, Supporting Information) and time‐resolved photoluminescence (TRPL) spectra (Figure S2b, Supporting Information) of the films with different morphologies demonstrate that the photoelectronic properties of these films can be different. In the PL spectrum, the wrinkled film exhibits a lower excitonic intensity compared with that of the flat one, but their excitonic bands are both at 550 nm. The TRPL spectra were fitted with a tri‐exponential function and the average lifetimes (*τ*_ave_) were ≈15 and 29 ns for the wrinkled film and flat film, respectively.

**Figure 2:**
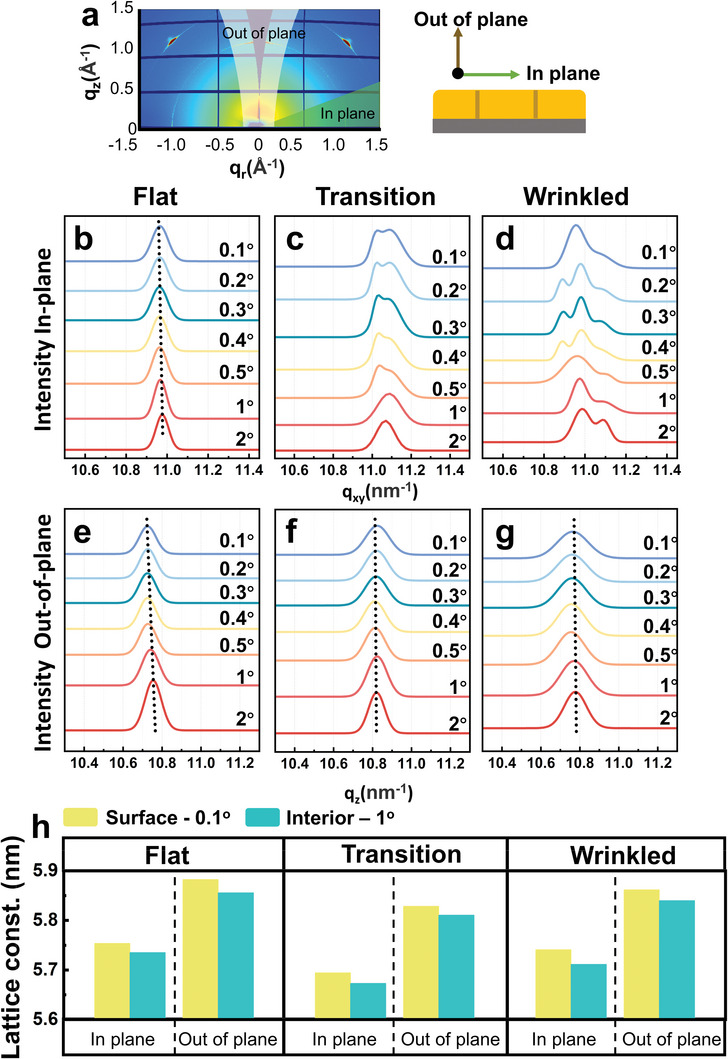
Residual strain distribution and evolution in the OIHP polycrystalline thin films. a) Illustration of the orientational integral ranges of the 2D GIWAXS pattern. b–d) 1D GIWAXS patterns along the in‐plane direction with different incident angles from 0.1° to 2° for b) the flat film, c) flat‐wrinkled transition film, and d) wrinkled film, respectively. e–g) 1D GIWAXS patterns along the out‐of‐plane direction with different incident angles from 0.1° to 2° for e) the flat film, f) flat‐wrinkled transition film, and g) wrinkled film, respectively. h) Calculated lattice constants of the crystals at the surface and interior of the flat film, flat‐wrinkled transition film and wrinkled thin film in the in‐plane direction and out‐of‐plane direction, respectively.

To explore the detailed residual strain distributions in the thin films with different morphologies, more ex situ GIWAXS characterizations were performed. Three kinds of film morphologies including flat one, flat‐wrinkled transition one and wrinkled one are selected. To quantitatively explain the relationship between the anti‐solvent dripping time and the film morphology, we fixed other factors, like the constitution of the precursor solution and the spin coating speed. The precursor solution consisted of N,N‐Dimethylformamide and Dimethyl sulfoxide (DMSO) with a volume ratio of 3:1, and the spin coating rate kept as 3000 rpm. In this scenario, the relationship between the anti‐solvent dripping time and the film morphology can be experimentally obtained and shown in Figure S3, Supporting Information. A series of incident angles ranging from 0.1° to 2° are adopted to study the depth‐dependent microstructure from the film surface down to its interior. The relationship between the incident angle and the penetration depth is shown in Figure S4 and Table S1, Supporting Information. The 1D GIWAXS patterns are obtained by the orientational integral of the 2D patterns in the in‐plane or out‐of‐plane directions (**Figure** **2**a). The in‐plane direction is parallel to the substrate, which reflects the (010) crystal planes (Figure 2b–d), while the out‐of‐plane direction is perpendicular to the substrate, which reflects the (001) crystal planes (Figure 2e–g). The Full Width at Half Maximum of the peak changes with the incident angle, as shown in Figure S5, Supporting Information.^[^ ^48^ ^]^

As shown in Figure 2b,e, with the increase of the incident angle, in the flat film, there is a shift trend of the MAPbBr_3_ crystalline peak to the positive *q* vector axis, no matter the direction of the orientation integral is in‐plane or out‐of‐plane. However, in Figure 2f,g, for the transition and wrinkled films, the peak position is almost constant, indicating that the strain keeps the same value in the out‐of‐plane direction.

In the in‐plane direction, except for the flat film, the transition film and wrinkled film has double or even triple peaks. The multi‐peaks indicate that the corresponding lattices are deformed with different strain states. In the flat film, the stress level is relatively low to generate a uniform strain, while in transition and wrinkled films, the stress levels could be higher to introduce different strain states. This split of the strain state also varies along the thickness direction. For the transition film, the multi‐peak is more obvious when closer to the surface. This is because the film‐substrate interface is still constrained by the substrate and maintains a relatively uniform strain state, which presents as a single peak in the 1D integral patterns. Nevertheless, the film surface is constrained less and the corresponding lattices have more deformation freedom, resulting in a more diverse strain states and multi‐peaks. On the other hand, for the wrinkled film, the multi‐peaks appear in all the film regions from the film‐substrate interface to the surface. This is because in the buckled area, both the top and the bottom of the film are free and with less constrains, leading to the split of the strain state across the entire thickness of the film.

Besides, the locations of the multi‐peaks also can be used to compare the strain magnitudes. Generally, in either the flat or transition film, the film‐substrate interface is constrained by the substrate to possess the highest compressive strain, while the film surface is free and in the lowest compressive strain state. The higher the compressive strain, the less the lattice constant, while the higher the *q* value. Therefore, with the increasing incident angle, the compressive strain will become higher, and the peak will shift to the right, which is consistent with the observation in Figure 2. In the wrinkled film, the total compressive strain should be the superposition of the global compressive strain and the bending strain. For this arch‐like shape, the bending strain should be compressive at the bottom and gradually become tensile to the top. Hence, the total compressive strain should also decrease from the bottom to the top, and this trend is the same as those in flat and transition films. Eventually, the 1D integral peak for the wrinkled film shifts to the right just as shown in Figure 2.

Because of the difference in grain size, the peak broadening is more apparent in the wrinkled film than that in the flat one. The average grain size of the wrinkled film is 239.3 ± 75.7 nm, while the average grain size of the flat film is 341.2 ± 140.5 nm as shown in Figure 1i.

According to the *q* values of the MAPbBr_3_ crystalline peak, the lattice constants of the different films were equal to 2*π*/*q* and illustrated in Figure 2h. All of the *q* values are corrected by considering the changing of incident angles^[^ ^49^ ^]^ (Table S2, Supporting Information). In the multi‐peak cases, the peak position was the weighted average by the peak area. Based on the GIWAXS data, all the lattice constants for different films are smaller than that in a strain‐free MAPbBr_3_ crystal, by referring to the experimentally determined lattice constants in the MAPbBr_3_ bulk single crystal^[^ ^50^ ^]^ (5.9266 Å) and the MAPbBr_3_ powder^[^ ^51^ ^]^ (5.92 Å) and theoretically estimated lattice constant via the Density Functional Theory simulation (5.96 Å).^[^ ^52^ ^]^ This comparison indicates that the residual strains in all the different thin films are compressive. Generally, for all the different films, the surface possesses a larger lattice constant than that of the interior, and the lattice constant in the out‐of‐plane direction is much larger than that in the in‐plane direction. Therefore, the residual compressive strain rises when the depth from the surface increases, meanwhile the crystal lattice in the in‐plane direction has a higher residual compressive strain when compared with that of in the out‐of‐plane direction. In addition, if the increase of the incident angle in the wrinkled part is taken into consideration, the X‐ray could penetrate more than the depth supposed. Since the deeper the material layer, the higher the compressive strain, the compressive strains near the film surface are overestimated, and the difference between the compressive strains of the top and the bottom parts is underestimated, which can strengthen the above conclusion.

It is worth noticing that, in the in‐plane direction, the flat‐wrinkled transition film has the smallest lattice constant and the highest residual compressive strain among all the thin films. Therefore, when dripping the anti‐solvent dripping in the early stage of the spin coating process, the residual compressive strain in the in‐plane direction originates and rises with the increase of the anti‐solvent dripping time, and the thin film remains flat. With the anti‐solvent dripping time continuously increasing to a critical point, the residual compressive strain in the in‐plane direction reaches its maximum value, and the thin film is about to camber with the flat‐wrinkled transition morphology. Eventually, when the anti‐solvent dripping time is later than the critical point, the excessive residual compressive strain in the in‐plane direction dramatically increases the instability of the film and makes it wrinkled to release the extra strain, resulting in a declined residual compressive strain in the in‐plane direction.

Besides the anti‐solvent dripping time, other process parameters, for example, the amount of the anti‐solvent and the chlorobenzene (CB) concentration in the anti‐solvent, were also considered as the factors to change the residual strain in the thin film. Figures S6 and S7, Supporting Information, illustrates that the morphology of the film changes from flat one to wrinkled one with the increase of the CB concentration and the amount of the same anti‐solvent, respectively. Therefore, the anti‐solvent dripping time and the constitution and amount of the anti‐solvent play significant roles in the formation of the thin film morphology, and the crystallization kinetics behind this phenomenon will be discussed as following.

### 2.2 Crystallization Speed‐Dependent Residual Strain in the OIHP Polycrystalline Thin Film

In general, the crystallization of the OIHP polycrystalline thin film is triggered by the evaporation of the precursor solvent, which can increase the concentration of the solute until to a saturated or supersaturated state.^[^ ^53^ ^]^ This process can either naturally proceed in any open system or be accelerated by an additional anti‐solvent. The anti‐solvent can dramatically extract the precursor solvent and make the precursor solution saturated or supersaturated in a short time.^[^ ^32^ ^]^ On the other hand, before the adding of the anti‐solvent, the precursor solution can also evaporate continuously, once it is dripped on the substrate. Therefore, the later anti‐solvent dripping time, the higher solute concentration of the precursor, when it encounters with the anti‐solvent. Different times of the anti‐solvent dripping result in different solute concentrations of the precursor to kick off different crystallization processes. The other two aforementioned parameters, the CB concentration and amount of the anti‐solvent can tune the acceleration effect of the anti‐solvent, leading to different crystallization processes as well. Hence, all three parameters can influence the crystallization process of the thin film and further result in different residual strains and film morphologies. Thus, it is rationally deduced that the crystallization process affected by the process parameters plays a significant in the distribution and evolution of the residual strain during the thin film formation.

**Figure 3:**
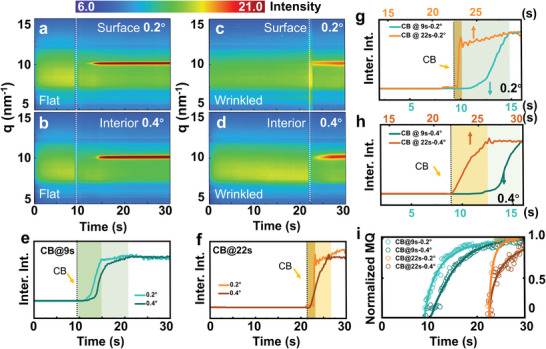
Crystallization processes with different anti‐solvent dripping times. a–d) In situ GIWAXS intensity mappings of the *q*‐time plane for thin film samples with different anti‐solvent dripping times: 9 s after the start of the spin coating with the incident angles of a) 0.2° (surface) and b) 0.4° (interior), respectively; 22 s after the start of the spin coating with the incident angles of c) 0.2° (surface) and d) 0.4° (interior), respectively. e) Comparison between the integrated intensities of the 1D patterns in (a) and (b). f) Comparison between the integrated intensities of the 1D patterns in (c) and (d). g,h) Comparisons between the integrated intensities of the 1D patterns of g) the surface and h) interior parts with different anti‐solvent dripping times. The time scales of the integrated intensity curves are aligned by the anti‐solvent dripping time. All the integrated intensity curves in (e–h) are integrated about the peak positions and background subtracted. i) Evolution of the material quantity against time. Solid lines are the fitted curves with the Avrami‐model.

To investigate the role of the crystallization kinetics in the residual strain evolution, 2D GIWAXS patterns were acquired 0.2 s per frame to observe of the real‐time crystallization process of the OIHP polycrystalline thin film during the anti‐solvent spin coating (Figure S8, Supporting Information). The in situ GIWAXS intensity mappings of the *q*‐time plane are for thin film samples with different anti‐solvent dripping times are displayed in **Figure** **3**a–d. The incident angles of the X‐ray are 0.2° and 0.4°, which can appropriately in situ monitor the crystallization process in the film surface and film interior, respectively. The anti‐solvent dripping times (marked with dashed lines) are chosen as the 9 s and 22 s after the start of the spin coating, which can lead to the typical flat film and wrinkled film, respectively.

For a thorough analysis, the entire fabrication process can be divided into three stages. The first stage is from the start time of the spin coating to the dripping time of the anti‐solvent. In this stage, both samples exhibit a broad scattering intensity (green color), which could originate from the colloids established in the precursor solution. The second stage starts with the anti‐solvent dripping and ends with the MAPbBr_3_ crystalline peak (red color) appearing. In this stage, the broad scattering intensity decreases at first, due to the sudden dilution of the precursor solution by the anti‐solvent dripping. Then, the broad scattering intensity resumes immediately and a noticeable signal (red color) appears at around 10 nm^−1^, which indicates the formation of the OIHP crystals. The final stage is from the appearance of the OIHP crystal peak to the time when it reaches the highest intensity, and at this time, the film is fully dried and crystallized.

To clearly observe the difference in crystallization kinetics between the two fabrication conditions, the integrated intensity curves of all the above pseudo‐color images are plotted in Figure 3e,f, where the intensities are normalized for comparison. When the anti‐solvent dripping time is the ninth second (Figure 3e), both the surface part and interior part of the thin film possess a distinct second stage, where the intensity curve ramps up slowly and the OIHP crystal peak has not appeared for several seconds after the dripping of the anti‐solvent. On the contrary, when the anti‐solvent dripping time is the 22^nd^ s (Figure 3f), the intensity curve rises instantly after the anti‐solvent dripping, and the second stage is quite short, especially in the surface part of the thin film. Apparently, the later dripping of the anti‐solvent can remarkably expedite the crystallization process. According to the basics of crystallization kinetics, the second stage is highly related to the induction period of the crystal nucleation. The start points of the second stage (i. e., the anti‐solvent dripping time) in different intensity curves of different samples are aligned to conveniently investigate the influence of the anti‐solvent dripping time on the crystallization process, as plotted in Figure 3g (surface) and h (interior). The different induction periods of the crystal nucleation can result in different kinetic processes of the crystallization. Usually, when the solvent keeps decreasing, the supersaturation state of a solution system can become the dissolved state, metastable state and labile state in sequence.^[^ ^54^ ^]^ In the dissolved state, the crystal nucleation unlikely happens. In the metastable state, if the supersaturation is low, the crystal can hardly nucleate without additional seed crystals, while if the supersaturation is high, the nucleation can spontaneously kick off after an induction period. Eventually, in the labile state, the supersaturation is ultra‐high, and the nucleation begins immediately without any induction period. In the anti‐solvent spin coating process, the anti‐solvent dripping time can determine the supersaturation state of the solution system, because the evaporation time of the precursor solvent before this moment could vary. When the anti‐solvent dripping time is the ninth second, both the surface part and interior part experience an obvious nucleation induction period, which means that the crystallization starts with a metastable state. On the contrary, the anti‐solvent dripping at 22^nd^ s results in the nucleation starts with a labile state. This can be the reason from the crystallization kinetics point of view why the experiment with the later anti‐solvent dripping possesses a faster crystallization.

Besides, the aforementioned results in Figure 3e,f also demonstrate the growth mode of the thin film, by the comparison of the integrated intensity curves representing its surface part and interior part. In both in situ GIWAXS experiments, the intensity curve of the film surface part rises earlier than that of the film interior part, which proves that the crystallization of the thin film starts from the surface and propagates downward to the substrate. In other words, rather than the traditional “bottom‐up” growth mode of the crystals in the CVD or electroplating method, the growth mode of the OIHP crystals in this work is “top‐down” via the anti‐solvent spin coating method. Additionally, the intensity curve of the film surface also reaches its maximum value earlier than that of the film interior, which indicates that the completion of the crystallization starts from the film surface part downward to the film interior part as well.

Moreover, in Figure 3e, with the anti‐solvent dripping at the ninth second, when the intensity of the film surface reaches the maximum value, the intensity of the film interior already becomes 60%–70% of the highest strength. The above percentage can be defined as the conversion percentage of the crystals, and the 100% value means the completion of the crystallization process. Apparently, this conversion percentage of the crystals is positively related with the fraction of the crystallized volume and reflects the progress of the crystallization. Interestingly, as shown in Figure 3f, with the anti‐solvent dripping at the 22^nd^ s, the intensity of the film interior is only 20%–30% of the highest strength, at the moment when the intensity of the film surface reaches the maximum value. This phenomenon means that although both the two film surface parts are completely crystallized, the two film interior parts possess different conversion percentages of the crystals, due to the different anti‐solvent dripping times. Generally, the later the anti‐solvent dripping, the less conversion percentages of the crystals in the film interior part, when the intensity of the film surface part reaches the maximum value.

For the flat and wrinkled films, the in‐plane lattice constants against time were shown in Figure S10, Supporting Information, and the values are normalized with the corresponding average value of the surface layer to clearly present the relative variations. It can be concluded that the lattice constants remain constant after the depletion of the solution. The lattice constant of the film surface (0.2°) is always larger than that of the interior (0.4°), which is consistent with the results of the ex situ GIWAXS in Figure 2.

Furthermore, the influence of the CB concentration in the anti‐solvent on the crystallization process was also investigated. The anti‐solvent with a lower CB concentration (25%) was dripped at the 22^nd^ s, and the X‐ray incident angle was set as 0.4° to be compared with the corresponding experiment with a 100% CB concentration in Figure 3d. The in situ GIWAXS intensity mapping of the *q*‐time plane for thin film samples with 25 vol % CB concentration of the anti‐solvent and its corresponding integration intensity curve are shown in Figure S11, Supporting Information. The results show that the anti‐solvent with a lower CB concentration also can trigger the crystallization process immediately, which means that this crystallization starts with a labile state as well. However, the lower CB concentration in the anti‐solvent can lead to a longer time to reach the maximum intensity, which manifests its influence on the kinetic process of the crystallization.

In order to estimate the material quantity (MQ), the azimuthal tube cut was performed, and the intensity is Lorentz‐corrected by a factor of sin *χ*. The area under each curve is integrated to obtain the MQ.^[^ ^55^, ^56^ ^]^ These areas can be analyzed over the entire spin coating process from the in situ GIWAXS data (Figure 3i). The Avrami model is used to quantitatively deduce the reaction speed and the crystal nucleation and growth topologies. Based on the fitting results (Table S3, Supporting Information), the earlier dripping of the anti‐solvent (CB @ 9s) results in a smaller *K* value, which indicates a lower reaction speed. Also, in both the films, the surface part has a higher reaction speed than that of the interior part. The *n* parameters are all around 1 in all fitting cases, indicating a similar high orientated crystalline growth mode.

As a summary, the anti‐solvent dripping time and the CB concentration in the anti‐solvent can influence the crystallization process of the OIHP polycrystalline thin film in two ways: 1) changing the time of the second stage, which is the induction period of the crystal nucleation; 2) changing the time of the third stage, where the intensity of the OIHP crystal peak rises from its appearance to its maximum value. These stages are both highly related to the crystallization kinetics in the form of the nucleation rate and crystal growth rate, which can influence the conversion percentage of the crystals in the thin film. Thus, we can define the crystallization speed as the changing rate of the conversion percentage of the crystals in the solution system. Therefore, the later dripping the anti‐solvent and the increase of the CB concentration in the anti‐solvent can both boost the crystallization speed, which enhances the residual compressive strain inside the thin film and eventually leads to the wrinkled morphology of the thin film.

### 2.3 Mechanical Model for the Crystallization Speed‐Dependent Residual Strain Evolution

**Figure 4:**
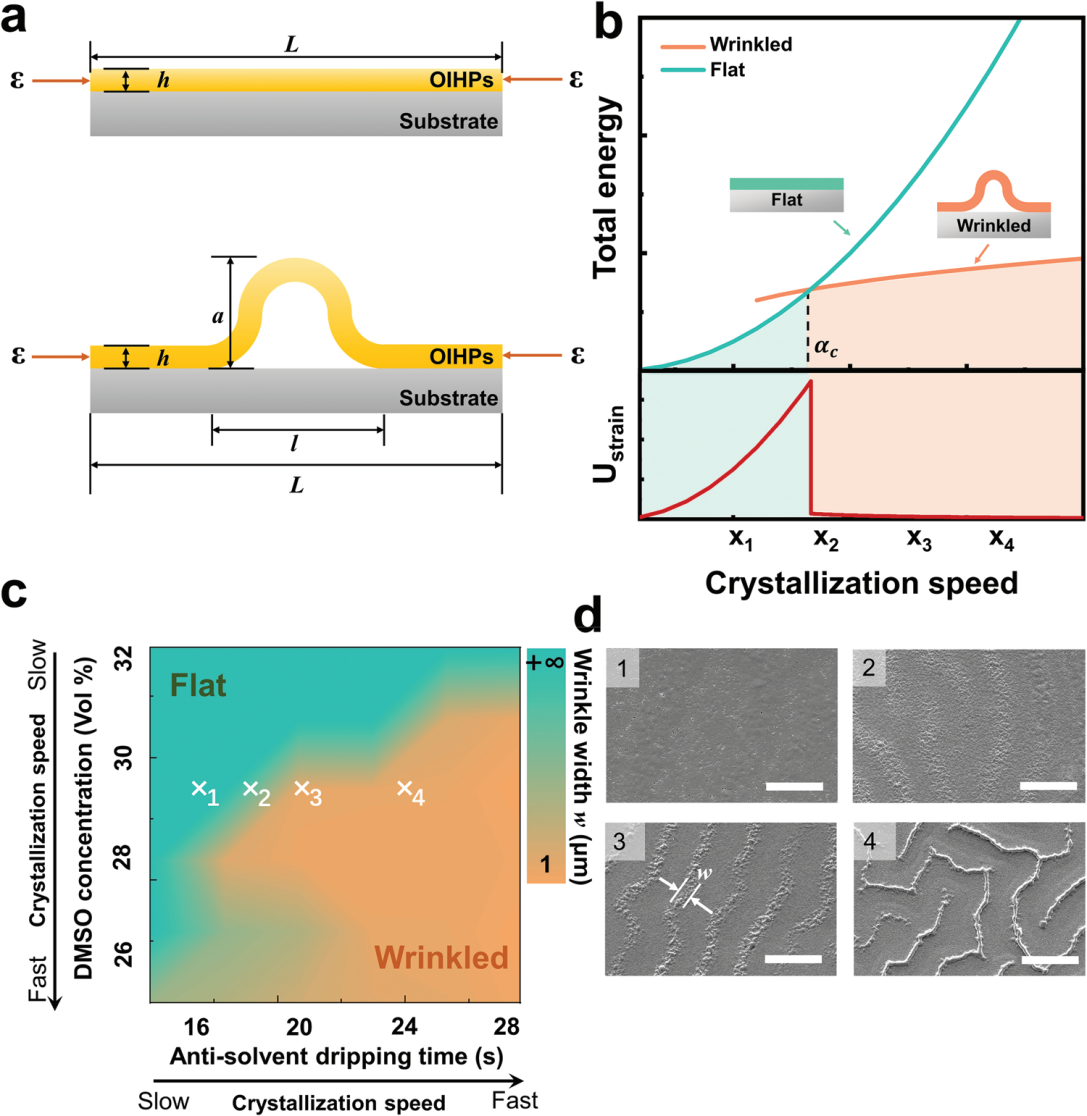
Mechanical model for the crystallization speed‐dependent residual strain evolution. a) Physical models of the OIHP polycrystalline thin films with flat morphology and wrinkled morphology, respectively. b) Comparison of the total energies for the flat and wrinkled OIHP polycrystalline thin films with varying crystallization speeds. c) Pseudo‐color mapping of the wrinkle width *w* with different DMSO concentrations in the precursor and anti‐solvent dripping times. d) SEM images of the film morphologies for the selected conditions in (c). All the scale bars are 200 µm.

To ascertain the relationships among the residual strain, crystallization speed and thin film morphology, we establish a mechanical model based on thin‐film mechanics^[^ ^57^, ^58^, ^59^, ^60^ ^]^ and crystallization kinetics. **Figure** **4**a illustrates the 1D physical models of the OIHP polycrystalline thin films with flat morphology and wrinkled morphology, respectively.

In this work, the energy minimization method^[^ ^57^, ^58^, ^59^, ^60^ ^]^ was adopted to establish the mechanical model to analyze the crystallization speed‐dependent residual strain evolution. The detailed calculation of the total energy is shown in note S1, Supporting Information. The upper part of Figure 4b illustrates the comparison of the total energy between the flat thin film and the wrinkled thin film on a glass substrate with different crystallization speeds. Naturally, for a certain crystallization speed, the film morphology should be the one with the lower total energy. When the crystallization speed is low, the flat morphology possesses lower total energy, and the film should be flat. As the crystallization speed increases to a critical crystallization speed *α*_c_, the two energy curves intersect as depicted in Figure 4b, and the film is at a flat‐wrinkled transition point. Then, when the crystallization speed is higher than the *α*_c_, the wrinkled morphology has lower total energy, and the equilibrated film should be wrinkled. Therefore, driven by the minimum process of the total energy, the film morphology changes from the flat one to the flat‐wrinkled transition one and finally to the wrinkled one, with an increasing crystallization speed.

Based on this mechanical model, the evolution of the residual compressive strain (denotes as *ε*_r_) during the morphology changing can be analyzed as well. Since the *U*_strain_ is proportional to the $\varepsilon_{r}^{2}$ (Detailed denotation and deduction are in note S1, Supporting Information), the evolution trend of the residual compressive strain is similar to that of the *U*_strain_ in the lower part of Figure 4b: 1) when the film is flat with a low *α*, the *ε*_r_ rises with the increasing *α*; 2) at the flat‐wrinkled transition point, the *ε*_r_ reaches its maximum values; 3) when the *α* is larger than the critical value, the film becomes wrinkled, and the *ε*_r_ is released to maintain a relatively low level. This calculated trend is highly consistent with the ex situ GIWAXS results in Figure 2g, in which the flat‐wrinkled transition film has the highest residual compressive strain and the lowest lattice constant along the in‐plane direction.

To further verify the aforementioned deduction, additional experiments with two varying parameters were conducted. The two parameters are the anti‐solvent dripping time and the DMSO concentration in the precursor, which are both common tunable parameters in the fabrication process and can influence the crystallization speed significantly.^[^ ^61^ ^]^ Figure 4c is a pseudo‐color mapping of the wrinkle width *w* with various combinations of the two above‐mentioned experimental parameters. Therefore, the crystallization speed increasing from the top‐left corner to the bottom‐right corner in Figure 4c, and the wrinkle width *w* changes from the infinity large (for the flat film) to the smaller values, which means that the morphology transits from the flat one to the obviously wrinkled one. Four representative points in Figure 3c are selected (x_1_–x_4_) to present the corresponding film morphologies with different parameter combinations (Figure 4d). Again, these experimental results are coincident with the calculated trend in Figure 4b very well, which validates the above mechanical model based on thin‐film mechanics and crystallization kinetics. Thus, both the experimental results and theoretical calculations demonstrate that when the crystallization process is accelerated, the thin film is easier to form a wrinkled morphology, which indicates a higher residual compressive strain inside the thin film.

## 3 Discussion

**Figure 5:**
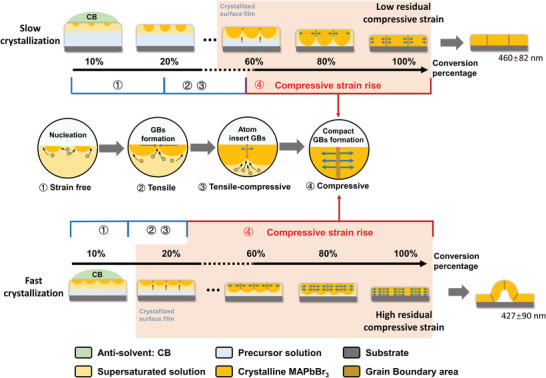
Schematic illustration of the mechanism for the crystallization speed‐dependent residual strain evolution. The orange color represents the MAPbBr_3_ crystals, the yellow color represents the supersaturated precursor solution mixed with the anti‐solvent, and the blue color represents the precursor solution not mixed with the ant‐solvent.

According to the above experimental results and theoretical calculations, the increase of the crystallization speed can enhance the residual compressive strain inside the film. However, the mechanism underlying the relationship between the crystallization speed and the residual compressive strain is still unclear. Thus, we attempt to propose a mechanism to explain this relationship based on the crystal growth process in the crystallization, as schematized in **Figure** **5**.

First, during the anti‐solvent spin coating, the crystallization of the OIHP polycrystalline thin film is a “downward” process triggered by the anti‐solvent dripping. The in situ GIWAXS results in Figure 3 imply that the anti‐solvent contacts the surface part of the precursor solution and extract precursor solvent,^[^ ^62^ ^]^ leading to a supersaturation state in this part instantly. Then, due to this supersaturation state, the crystal nucleates in the surface part prior to the interior part of the precursor solution, and grow both along the in‐plane and out‐of‐plane directions. Because the major nucleation occurs in the surface part, a completely crystallized surface layer is formed first along the in‐plane direction, while the crystal growth still proceeds along the out‐of‐plane direction. Thus, this process is named as the “downward” crystallization.^[^ ^32^, ^33^, ^34^, ^35^ ^]^ Notably, the crystallization speed affected by the degree of the supersaturation can result in differences in the crystallization process. As shown in the top part of Figure 5, when the crystallization speed is low, it takes more time to form the completely crystallized surface layer. Therefore, when the crystallization of the surface layer is just completed, the crystal growth along the out‐of‐plane direction is relatively sufficient, resulting in a 60%–70% conversion percentage of the crystals in the interior part, as shown in Figure 3e. However, when the crystallization speed is high as presented in the bottom part of Figure 5, the crystallized surface layer completes within a very short time, and the conversion percentage of the crystals in the interior part is only 20%–30% at that moment.

Second, the residual compressive strain in the OIHP polycrystalline thin film could originate from the excessive absorption of the free atoms in the precursor by the crystallized surface layer. For all kinds of thin films, especially polymer thin films, the residual strain can be observed after a similar “downward” solidification or crystallization process in certain fabrication conditions. There are possible theories for the formation of the residual strain in the film,^[^ ^63^, ^64^ ^]^ with different resultant residual strain distribution inside the film. In the first theory, the residual strain originates from the mismatch between the layers solidified at different moments. In a “downward” process, when the surface layer is completely solidified, there should be a certain amount of solution between the solidified surface layer and the substrate. At this moment, due to this in‐between solution, there is no strain in the solidified surface layer, which is constrained by the substrate at its edge. Subsequently, the volume shrinkage of the in‐between solution during its solidification caused by a phase transition can result in a tensile strain in this later solidified layer but a compressive strain in the surface layer along the in‐plane direction. Obviously, this distribution of the residual strain is inconsistent with the ex situ GIWAXS results in our study, and the first theory is not applicable to this work. On the other side, in the second theory, the residual strain is a result of the excessive absorption of the precursor free atoms by the solidified surface layer. In the solidification process, the formation of the solidified surface film causes a chemical potential gradient in the precursor solution, leading to the diffusion of the free solute atoms to the solidified surface layer. By absorbing the excessive free atoms from the precursor solution, the solidified surface layer gradually expands in volume. With the constraint by the substrate at its edge, the residual compressive strain originates along the in‐plane direction. Therefore, the residual compressive strain in the OIHP polycrystalline thin film originates and gradually rises with the solidification of the entire film (A detailed analysis is in note S2, Supporting Information). Because the film surface is less constrained, the residual compression strain along the in‐plane direction gradually increases from the surface to the interior, which is highly consistent with the ex situ GIWAXS results in our study. Hence, the second theory is quite applicable to the OIHP polycrystalline thin film in this work. Considering the residual strain distribution inside films in our study, the origin of the residual strain in OIHP polycrystalline thin film should be the excessive absorption of the precursor free atoms by the surface layer.^[^ ^64^ ^]^ During the solidification process, the formation of the crystallized surface layer causes a chemical potential gradient in the precursor solution, leading to the diffusion of the free solute atoms to the crystallized surface layer. Then, the excessive free atoms from the precursor solution could be absorbed by the crystallized surface layer, which tends to expand in volume. For a film growing on a finite substrate, the edge of the thin film dried faster than the inner part of the film, and then the entire solidified surface layer was anchored with the substrate at its edge. This is because that when the solidified surface layer forms, the precursor solution at the edge still can contact with atmosphere directly in the lateral direction, while the precursor solution in the inner part is isolated from atmosphere. Therefore, the precursor solution at the edge will evaporate faster and dry first. Once the edge is constrained, the film will develop a lateral or in‐plane strain during its subsequent crystallization process.^[^ ^51^ ^]^ Because the film surface is less constrained, the residual compression strain along the in‐plane direction gradually increases from the surface to the interior, which is highly consistent with the ex situ GIWAXS results in Figure 2.

Finally, the grain boundaries (GBs) in the crystallized surface layer are the main sites to absorb extra free atoms from the precursor solution, and the number of the absorbed free atoms can be influenced by the crystallization speed, further resulting in different residual compressive strain levels. Four steps of the crystal growth and GB formation in the OIHP polycrystalline thin film are depicted in the middle part of Figure 5. In Step 1, due to the dripping of the anti‐solvent, the crystal nuclei form and grow like independent islands in the surface part of the thin film. Obviously, there is no strain generated. In Step 2, each independent nucleus grows and form GBs due to the energy minimization, which generate a tensile strain inside the grains. In Step 3, because the chemical potential of an atom is lower when being placed in the GB region, the free atoms in the precursor solution are inserted into the GBs.^[^ ^65^ ^]^ With the increasing number of the inserted free atoms, the residual strain inside the grains changes from a tensile one to a tensile‐compressive transition one and finally to a compressive one.^[^ ^66^ ^]^ In Step 4, the entire thin film is completely crystallized, and the residual strain is eventually determined. Hence, the number of the inserted free atoms into the GBs is the key issue in the evolution of the residual strain. Different from the traditional CVD method or electroplating method, where the raw materials are continuously supplied at a constant speed, in the anti‐solvent spin coating method, a limited precursor solution as the raw material is provided to fabricate the OIHP polycrystalline thin film. Therefore, the number of the free atoms in the precursor solution at the moment when the crystallized surface layer is just formed determines the upper limit of the number of the inserted free atoms into the GBs. According to the in situ GIWAXS results in Figure 3, when the formation of the crystallized surface layer just completes, in the experiment with the higher crystallization speed, the conversion percentage of the crystals in the interior part is only 20%–30%, indicating the more free atoms in the precursor solution than those with the lower crystallization speed. Eventually, the more atoms inserted into the GBs lead to a higher residual compressive strain with the faster crystallization process. On the contrary, with a slow crystallization speed, only a small amount of remaining precursor solution exists in the system when the crystallized surface layer is formed. Therefore, few free atoms can fill in the GBs which form a lower residual compressive strain in the film. In addition, with a higher crystallization speed, the grain size also becomes smaller and the region of the GB expands (Figure 1i–h), which provides more sites to absorb free atoms and further intensifies the tendency of the thin film to expand. Thus, the mechanism underlying the relationship between the crystallization speed and the residual compressive strain is revealed, and the general trend is that the faster the crystallization process, the more free atoms inserted into the GBs, and the higher the residual compressive strain in the thin film.

Based on the above experimental results and mechanism analysis via combining thin film mechanics and crystallization kinetics, we revealed the formation and evolution of the compressive strain in the OIHP polycrystalline thin films during crystallization and suggested that the crystallization speed of the film could be the key influencing factor. In practical, the residual strain inside the OIHP polycrystalline thin film can considerably influence the energy conversion efficiency and stability of the device. An appropriate compressive strain can improve the energy conversion efficiency and stability of the thin film perovskite solar cell. However, an excessive compressive strain can make the film wrinkled or even fractured, which will significantly deteriorate the device performance. Base on this work, researchers could introduce a suitable compressive strain level via tuning the crystallization speed to enhance the device performance but without any unfavorable morphology, for examples, wrinkles or cracks. Therefore, leveraging on this study, the researchers could have gained a practical approach to optimize the residual strain in their films effectively and efficiently and further important the performance of their device.

## 4 Conclusion

In summary, we investigate the residual strain distribution and evolution of the OIHP polycrystalline thin films during the anti‐solvent spin coating process and reveal that the crystallization speed is an important factor to influence the residual strain inside the film and the film morphology. Based on the GIWAXS experiments and the mechanical model, we illustrate the relationship between the crystallization speed and the residual strain evolution, which can determine the final film morphology as well. With the increase of the crystallization speed, the residual compressive strain inside the OIHP polycrystalline thin film continuously increases and forms a residual strain gradient from the film surface to the film interior. There is a critical point of the crystallization speed, where the residual compressive strain reaches the maximum value, and the film morphology changes from the flat one to the wrinkled one. From the perspective of crystallization kinetics, in the “downward” process triggered by the anti‐solvent dripping, the crystallization speed can influence the number of the excessive absorbed free atoms from the precursor solution by the crystallized surface layer, further resulting in different residual compressive strain levels. The GBs in the crystallized surface layer are the main absorption sites of the free atoms. Normally, the faster the crystallization process, the more free atoms inserted into the GBs, and the higher the residual compressive strain in the OIHP polycrystalline thin film. Based on advanced characterization techniques, the mechanical model and crystallization kinetics, this work reveals a deeper and more comprehensive understanding of the residual strain evolution during the anti‐solvent spin coating process in the OIHP polycrystalline thin films and provides important guidelines for the residual strain‐related strain engineering, morphology control and photo‐electronic performance enhancement of the thin films.

## Conflict of Interest

The authors declare no conflict of interest.

## Supporting information

Supporting Information

## Data Availability

The data that support the findings of this study are available from the corresponding author upon reasonable request.
