# Supplementary material for: Residual Strain Evolution Induced by Crystallization Kinetics During Anti‐Solvent Spin Coating in Organic–Inorganic Hybrid Perovskite
Source: Adv Sci (Weinh). 2023 Apr 25;10(18):2205986. doi: 10.1002/advs.202205986 (PMC10288237; doi:10.1002/advs.202205986)
Supplement: Supplementary file 1 — Supporting Information [file ADVS-10-2205986-s001.pdf]

ADVANCED SCIENCE
Open Access

Supporting Information

for *Adv. Sci.*, DOI 10.1002/advs.202205986

Residual Strain Evolution Induced by Crystallization Kinetics During Anti-Solvent Spin Coating in Organic–Inorganic Hybrid Perovskite

Y. Sun, Q. Yao, W. Xing, H. Jiang, Y. Li, W. Xiong, W. Zhu and Y. Zheng**

Supporting Information

Residual strain evolution induced by crystallization kinetics during anti-solvent spin coating in organic-inorganic hybrid perovskite

Yijing Sun[1,2,3], Qianqian Yao[1,2,3], Weiwei Xing[1,2,3], He Jiang[1,2,3], Yingbo Li[1,2,3], Weiming Xiong[1,2,3], Wenpeng Zhu[1,2,3,], Yue Zheng[1,2,3,*]*

Materials and Methods

Thin film fabrication. The $MAPbBr_3$ polycrystalline thin film was synthesized by the one-step solution method on a glass substrate. The glass substrate was sequentially washed with isopropanol, acetone, distilled water and ethanol and gently dried with a nitrogen flow afterward. The spin coating deposition of $MAPbBr_3$ film by one-step solution method [1] was performed inside a nitrogen-filled glove box with < 0.01 ppm O_2 and H_2O. The MABr (> 99.5%, Lumtec) and $PbBr_2$ (99.999%, Aladdin) with a molar ratio of 1:1 were dissolved in a mixture solution of N,N-Dimethylformamide (>99.7%, Acros) and DMSO with a volume ratio of 3:1 to form the precursor solution (40 wt.%). The precursor solution was stirred at 60 °C for 2 h and then filtered through a 220 nm PTFE filter. The $MAPbBr_3$ film was deposited on the glass substrate by spinning 70 μL of the filtered solution at 3000 rpm for 25 s. During the spin coating process, 120 μL of anti-solvent solution was dripped onto the spinning layer at the different dripping times of spin coating. In most experiments, the anti-solvent was pure chlorobenzene. In experiments with various anti-solvent concentrations, the concentration of the anti-solvent solution was tuned by mixing the chlorobenzene and ethyl alcohol in different volume fractions from 0-100 vol%, with the same anti-solvent dripping time.

In experiments with various anti-solvent amount, the anti-solvent was pure chlorobenzene, and its volume was changed from 5μL to 120μL, with the same anti-solvent dripping time. In the experiments with the anti-solvent of a lower chlorobenzene concentration, we chose ethyl alcohol mixed with chlorobenzene as the anti-solvent, to tune the crystallization speed

of the $MAPbBr_3$ film. On the one hand, the ethyl alcohol could dilute the chlorobenzene concentration in the anti-solvent solution, to reduce its effect on accelerating the crystallization of the perovskite film. On the other hand, the ethyl alcohol could slightly decompose the perovskite crystalline to further slowdown the crystallization of the perovskite film [2-6].

Crystal structure, orientation and phase composition characterizations.

The ex-situ GIWAXS experiments were carried out at beamline BL14B1 (BL17B1) in Shanghai Synchrotron Radiation Facility (SSRF). The monochromated energy of the X-ray source was 10 keV. The data were obtained with a PILATUS detector with a resolution of 1475 by 1679 pixels (253.7 mm by 288.18 mm). The sample-to-detector distance was 400 mm, and a calibration was conducted with a LaB6 sample. The X-ray wavelength was 1.2378 Å with a beam size of 150 × 80 μm^2. The incidence angle was set at a range from 0.1° to 2° for the penetration depth 2.9-1000 nm. The exposure time was set as 2 s. All of the ex-situ GIWAXS experiments were conducted under the protection of a N_2 gas flow.

The in-situ GIWAXS experiments were carried out at beamline BL14B1 (BL17B1) in Shanghai Synchrotron Radiation Facility (SSRF). The monochromated energy of the X-ray source was 10 keV. The data were obtained from a planar CCD detector with a resolution of 3072 by 3072 pixels (225 mm by 225 mm) and a PILATUS detector with a resolution of 1475 by 1679 pixels (253.7 mm by 288.18 mm). The sample-to-detector

distance was 350 mm, and a calibration was conducted with a LaB6 sample. The X-ray wavelength was 1.2378 Å, and the incidence angle was set at 0.2° and 0.4° for the penetration depth of 9-200 nm. The exposure time for each frame was set as 0.2 s. The spin coating was conducted under the protection of a N_2 gas flow. The spin coating system consisted of a spin coater and a motorized syringe, and they could be remotely controlled to conduct spin coating and adding the anti-solvent, respectively. After the perovskite precursor was dropped on the substrate, with the start of the spin coating process, the GIWAXS measurement could be triggered simultaneously, which was controlled by a computer program. The anti-solvent was added on the spinning film at a designated timing during the whole 30-second spinning process at 3000 rpm. The 2D GIXRD patterns were analyzed using the pyFAI, GIXSGUI and GIWAXS Tool software. [7]

For the in-situ GIWAXS experiments, the sample kept spinning, and the X-ray beam damage could be shared by different positions to mitigate the severe damage. For the ex-situ ones, to examine any difference in lattice along the film thickness direction, we fixed the sample and the X-ray beam to eliminate potential errors due to the moving sample or the varying position. In each experiment, we used the minimum incident angle to conduct the first exposure, and then gradually increased the incident angle to the maximum one. In this case, the material in each depth was fresh with no exposure history, until the X-ray beam could just reach this depth with a certain incident angle. Before the characterization in a certain depth, this strategy could reduce the unnecessary X-ray beam damage to the

material in this depth. Besides, the energy of the X-ray beam was set as 10 keV to minimize the damage from the X-ray. [8]

Other characterizations. The SEM images were taken by a Quanta 250FEG (FEI) with an acceleration voltage of 5 kV. PL spectra were obtained by using an FLS980 Spectrometer (Edinburgh, Germany) with an excitation source of 365 nm lasing wavelength. Minority carrier lifetimes were determined by a WT-2000 (SEMILAB, Hungary) with a laser wavelength of 405 nm via the μ-PCD (Microwave photoconductivity decay) method.

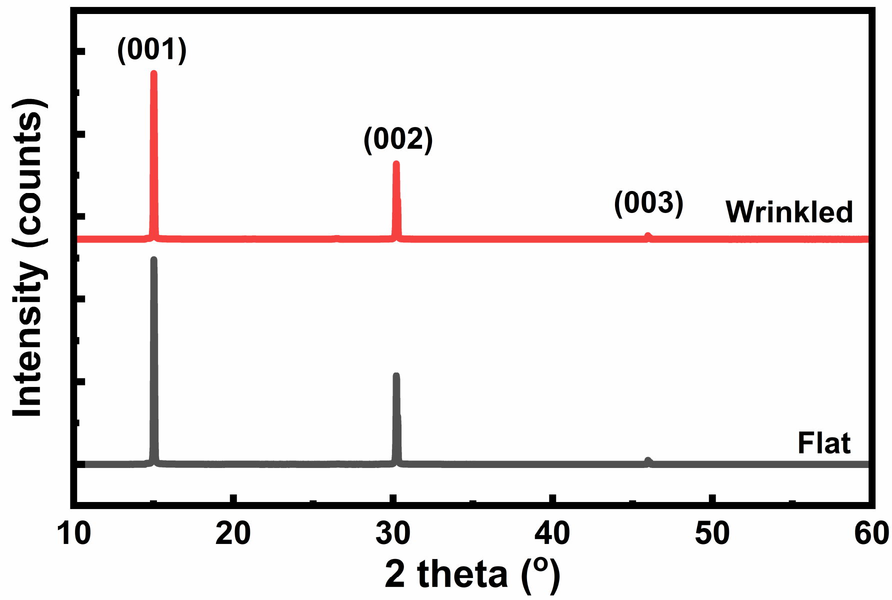

Figure S1. XRD results of the wrinkled and flat $MAPbBr_3$ thin films.

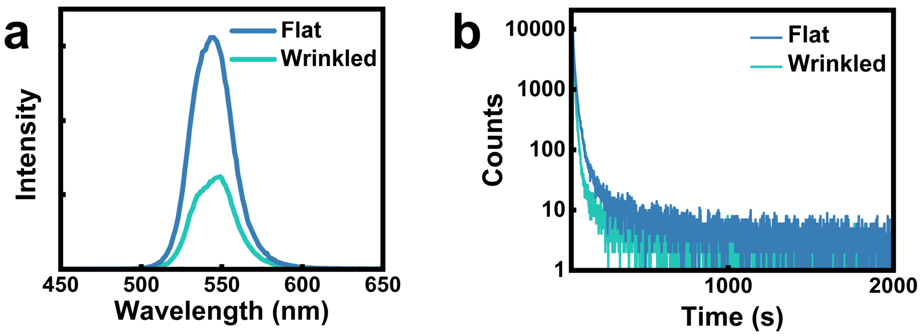

Figure S2. a) Steady-state photoluminescence (PL) spectra of the flat and wrinkled films.

b) Time-resolved photoluminescence (TRPL) spectra of the flat and wrinkled films.

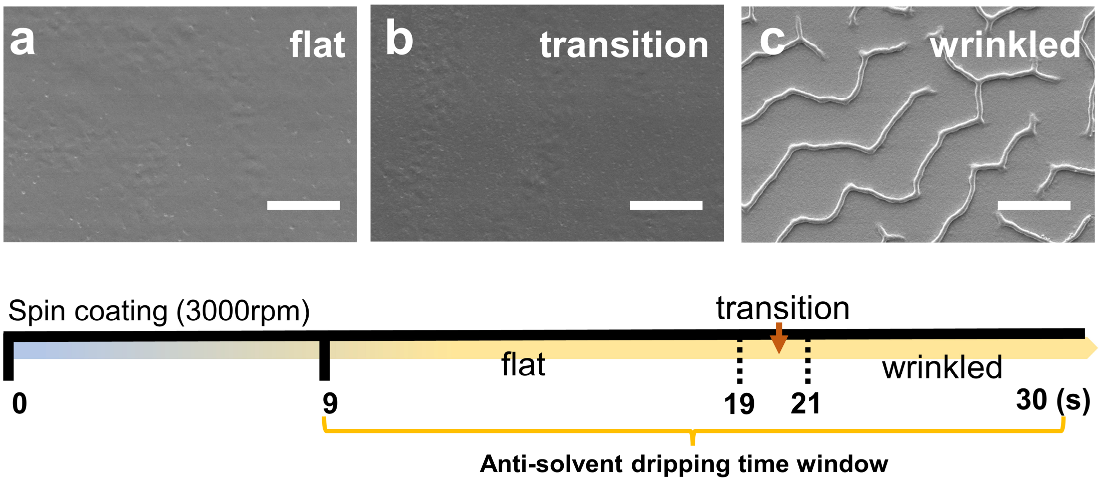

Figure S3. Relationship between the anti-solvent dripping time and the film morphology. SEM images of (a) the flat, (b) flat-wrinkled transition and (c) wrinkled OIHP polycrystalline films. All the scale bars are 200 nm. Other fixed factors: the precursor solution consists of DMF and DMSO with a volume ratio of 3:1, and the spin coating rate keeps as 3000 rpm.

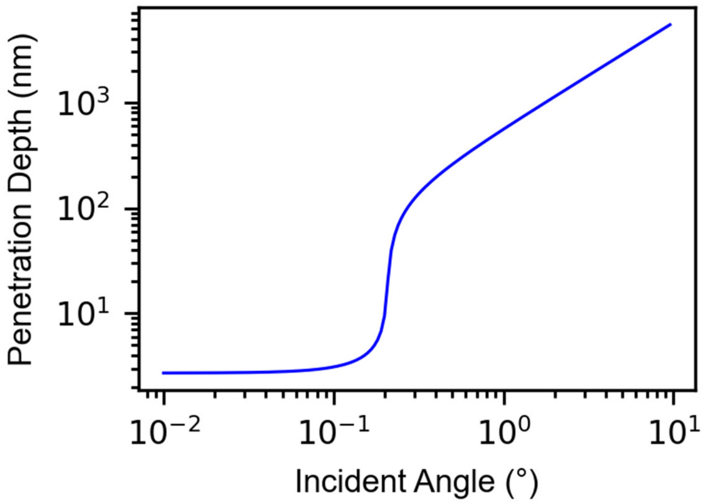

Figure S4. Relationship between the penetration depth and the incident angle.

Table S1. Penetration depths in ex-situ GIWAXS experiments.

Incident angle (º)	Penetration depth (nm)
0.1	2.9
0.2	9
0.3	124.8
0.4	193
0.5	254
1	552
2	1106

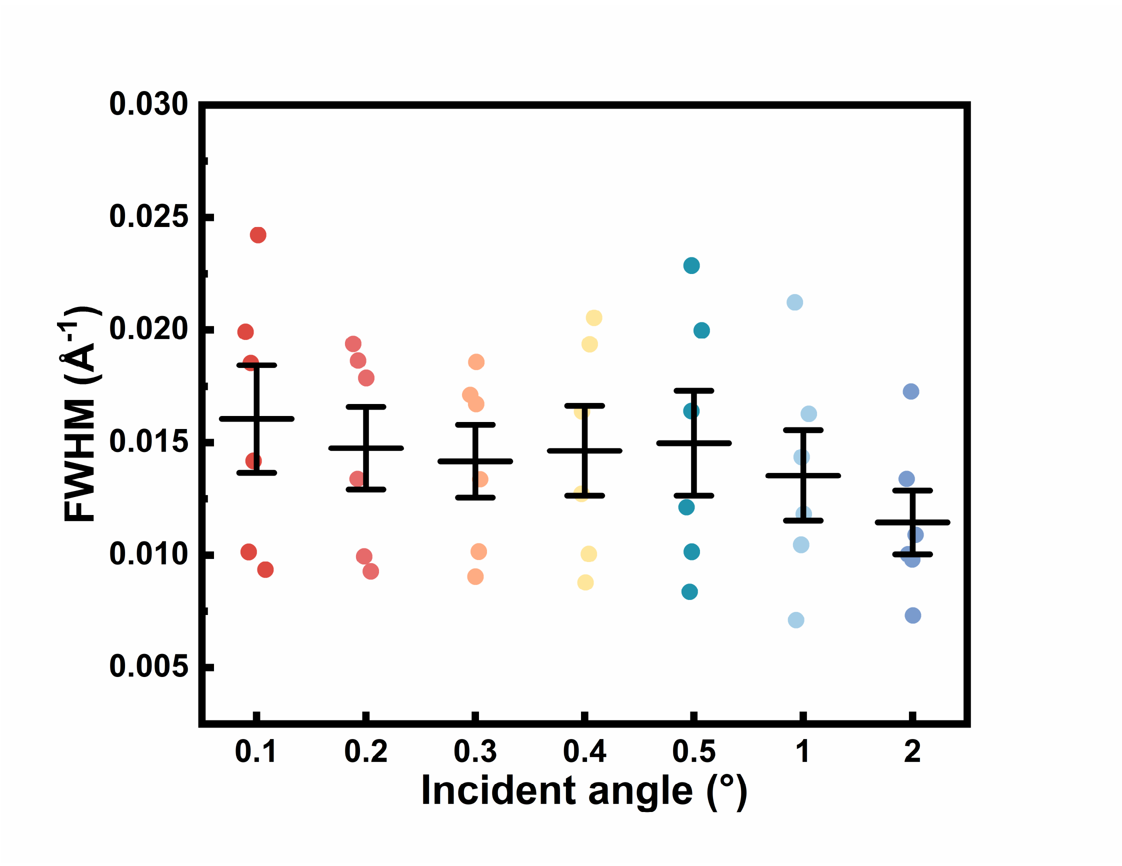

Figure S5. The statistical results of the FWHM with different incident angles in ex situ GIWAXS experiments.

Table S2. Comparison of the calculated lattice constants before and after the correction by considering the influence of the changing incident angle.

Orientation	Film type	Incident angle	Peak position q ($Å^{-1}$)		Lattice spacing (Å)	
			Before	After	Before	After
In plane	Flat	0.1	1.09652	1.09203	5.73011	5.75369
		1	1.09668	1.09556	5.72928	5.73516
	Transition	0.1	1.10791	1.25108	5.67123	5.69432
		1	1.10866	1.10753	5.66737	5.67313
	Wrinkled	0.1	1.09898	1.09449	5.71741	5.74088
		1	1.10124	1.10012	5.70564	5.71148
Out of plane	Flat	0.1	1.07261	1.06812	5.85784	5.88249
		1	1.0741	1.07298	5.84972	5.85584
	Transition	0.1	1.08248	1.07799	5.80444	5.82863
		1	1.08240	1.08127	5.80486	5.81089
	Wrinkled	0.1	1.07636	1.07187	5.83744	5.86191
		1	1.07700	1.07588	5.84227	5.84005

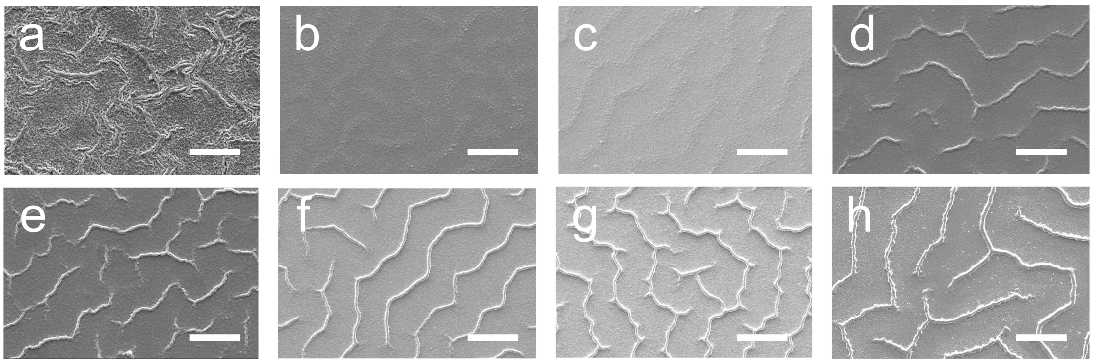

Figure S6. SEM images of the OIHP polycrystalline films fabricated with anti-solvents with different chlorobenzene concentrations (Vol %): (a) 0 %, (b) 12.5 %, (c) 25 %, (d) 37.5 %, (e) 50 %, (f) 62.5 %, (g) 75 % and (h) 100 %. The scale bars are 200 nm.

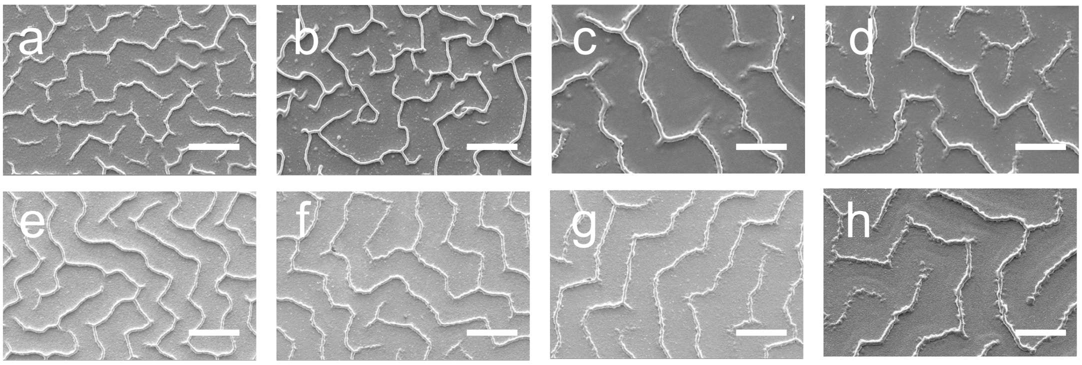

Figure S7. SEM images of the OIHP polycrystalline films fabricated with different amounts of the same anti-solvent (100% chlorobenzene concentration): (a) 5 μL, (b) 10 μL, (c) 20 μL, (d) 30 μL, (e) 50 μL, (f) 100 μL, (g) 150 μL and (h) 200 μL. The scale bars are 200 nm.

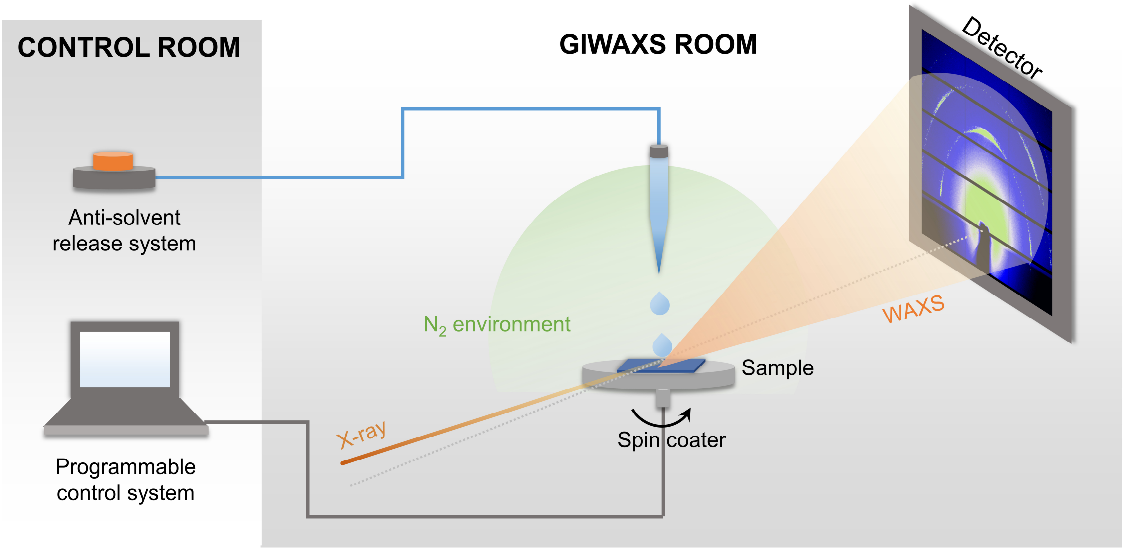

Figure S8. Equipment setup for the in situ GIWAXS during anti-solvent spin coating process.

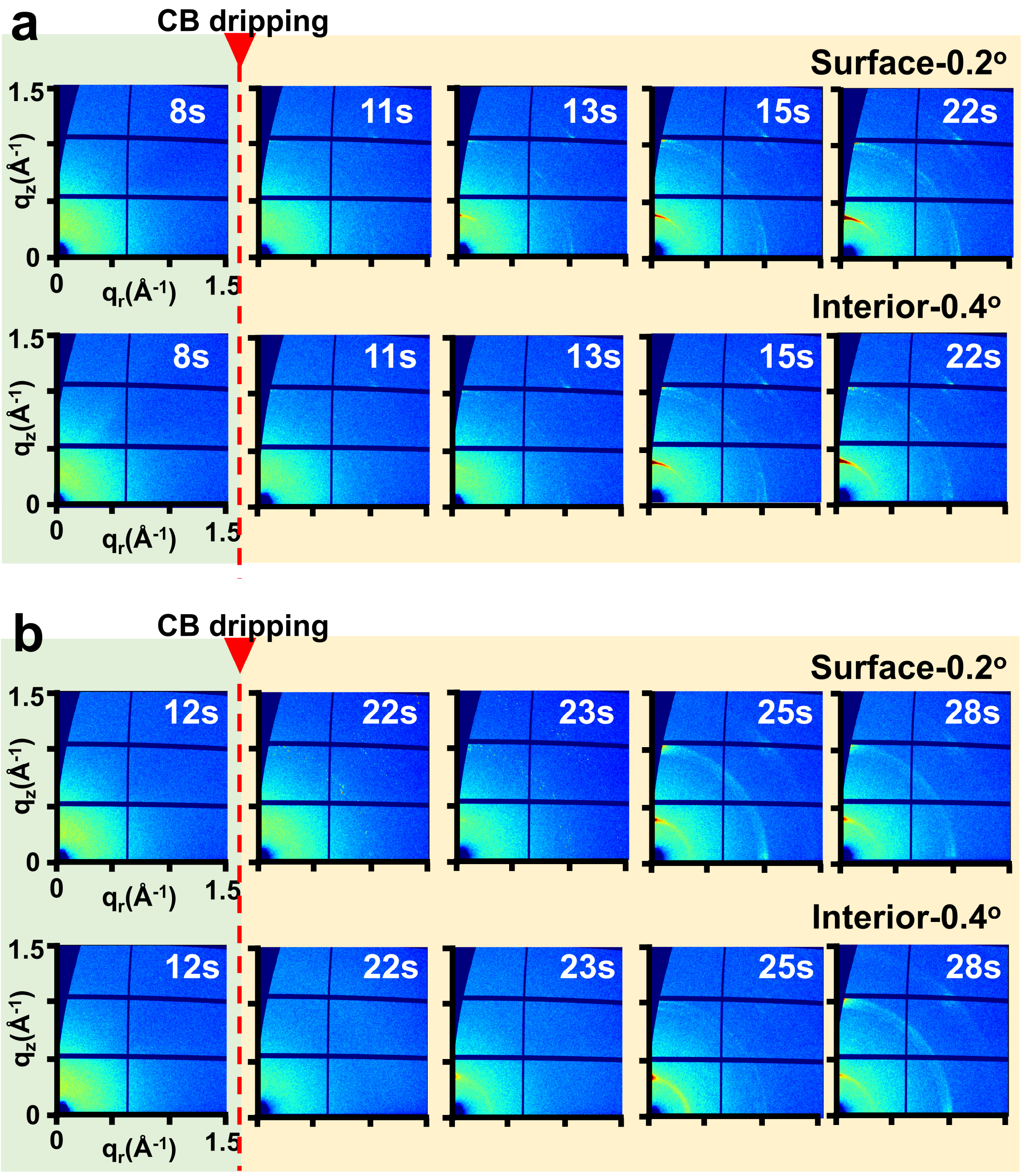

Figure S9. In-situ 2D GIWAXS patterns for (a) the flat and (b) wrinkled films.

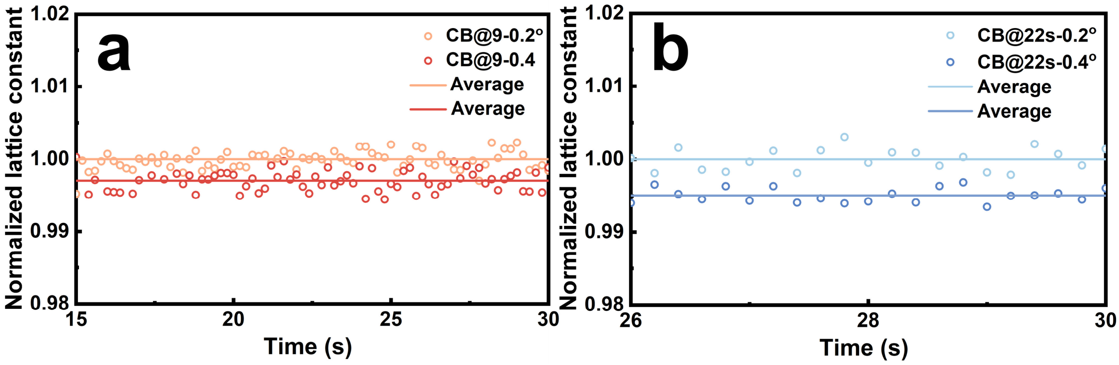

Figure S10. Normalized lattice constants of the (a) flat and (b) wrinkled films against the spin coating time.

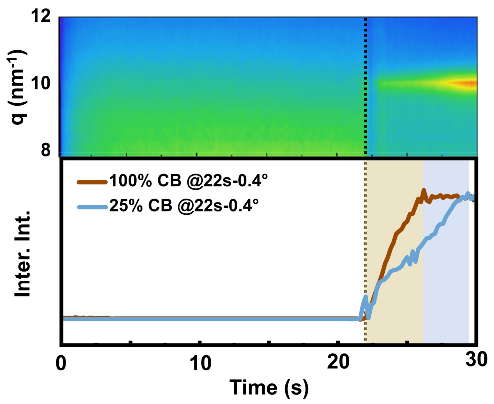

Figure S11. In-situ GIWAXS intensity mapping of the *q*-time plane for thin film samples with 25 vol % chlorobenzene concentration of the anti-solvent and its corresponding integration intensity curve (compared with the curve in Figure 3f). The integrated intensity curve is integrated about the peak positions and background subtracted.

Table S3. K and n parameters fitted in the Avrami model.

Film type	Position	K	n
**Flat (CB @ 9s)**	Surface	0.412	0.757
	Interior	0.144	1.130
**Wrinkled (CB@22s)**	Surface	1.214	0.756
	Interior	0.522	0.658

Supplementary note 1

The total energy calculation. If there is a single wrinkle on the film, the L denotes the length of the substrate, and the substrate is rigid. The h denotes the thickness of the thin film. For the wrinkled film, the l and a denote the width and height of a wrinkle, respectively. Based on the observation of all the experiments, the overall trend is that the higher the crystallization speed, the more obvious the wrinkles. For a film with obvious wrinkles, if the constraint from the substrate is removed, the film will be in a strain-free status and expand to a larger size. If we assume the strain-free length of the film is L_0, the more obvious the wrinkles, the larger the L_0. Then, the ε can denote as the nominal strain resulting from the crystallization process,

$$\varepsilon = \left|\frac{L - L_0}{L_0}\right|$$

In this work, the L_0 is always larger than the L, and the ε is compressive along the in-plane direction. From the equation deduction point of view, this nominal strain is similar to the concept of prestrain in previous studies on flexible electronics [9-12]. If there are multiple wrinkles on the film, the L denotes the length of one wrinkled unit, equal to the distance between two neighbor wrinkles.

According to the results of the in-situ GIWAXS experiments and morphology observations, we can assume the nominal strain is a function of the crystallization speed,

$$\varepsilon = f(\alpha)$$

where the α is the crystallization speed. To simplify the physical model, the relationship between the ε and α can be linear,

$$\varepsilon = C\alpha$$

where the C is a linear constant. The concept of the α is similar to the K parameter in the Avrami model, which represents the reaction speed. Therefore, they can be approximately equal to each other ($\alpha \approx K$).

The total energy in the thin film is the sum of the membrane strain energy (U_{strain}), interface energy ($U_{interface}$) and bending energy ($U_{bending}$). The membrane strain energy reflects the overall residual compressive strain of the thin film that is proportional to the average variation of the lattice constant, the interface energy is estimated from the bonding area between the film and substrate, and the bending energy is an additional term to describe the bending status of the wrinkles if applicable.

The total energy of the flat film consists of membrane strain energy and interface energy, which could be written as,

$$U_f = U_{strain} + U_{interface} = \frac{1}{2}EhL\varepsilon^2 - \gamma L$$

where, *E* is Young's modulus of the film, γ is the work of adhesion between the film and substrate.

The total energy of the wrinkled film consists of membrane strain energy, bending energy and interface energy, which could be written as,

$$U_w = U_{strain} + U_{bending} + U_{interface} = \frac{1}{2}EhL(\frac{\pi^2 a^2}{4Ll} - \varepsilon)^2 + \frac{\pi^4 E(2h)^3 a^2}{12l^3} - \gamma(L - l)$$

where, l and a denote the width and height of the wrinkle.

If $U_f = U_w$, the film reaches the critical nominal strain ε_c, and the film is in a flat-wrinkled transition state, whose crystallization speed is also critical value $\alpha_c(\approx K_c)$. The equation to calculate the critical nominal strain is $\varepsilon_c = 5(\frac{\pi}{8\sqrt{3}}\frac{\gamma}{EL})^{2/5} \approx 2.76(\frac{\gamma}{EL})^{2/5}$. In our case, by substituting E = 5 GPa, L = 10 μm and γ = 0.9 N/m, we can obtain the theoretical $\varepsilon_{c,th.}$ = 3.5%. Based on the GIWAXS data in Table S2 of the transition film, its average strain can be calculated as $\varepsilon_{c,ex.}$ ≈ 4.0%, which is the experimental critical nominal strain. As a preliminary theoretical model with several simplifying assumptions, this accuracy is acceptable.

The membrane energy calculation. Assume the overall/average residual compressive strain of the thin film is ε_r, and $U_{strain} = \frac{1}{2}EhL{\varepsilon_r}^2$ for both the flat film and wrinkled film, where E is the Young's modulus of the film. U_{strain} can be also expressed as below,

$$\begin{cases} U_{strain} = \frac{1}{2}EhL\varepsilon^2 \ (\alpha < \alpha_c, for\ flat\ film) \\ U_{strain} = \frac{1}{2}EhL(\frac{\pi^2 a^2}{4Ll} - \varepsilon)^2 \ (\alpha > \alpha_c, for\ wrinkled\ film) \end{cases}$$

The critical crystallization speed estimation.

In the flat film, the nominal strain ε is equal to the residual strain ε_r, which can be calculated with the GIWAXS data in Table S2. Therefore, we can combine the K parameter in the Avrami model and the above GIWAXS data of the flat film to estimate the linear constant C. Because of the ε and $\alpha(\approx K)$ in this model are overall parameters, we can average the parameter values for the surface and interior respectively to represent the overall parameters of the film. For the flat film, the average value of the K parameter is 0.278, and the average value of the ε parameter is 2.97%. Hence, the linear constant C can be estimated as $C \approx \frac{\bar{\varepsilon}_{flat}}{\bar{K}_{flat}} = 0.1068.$

Then, we can evaluate the theoretical critical crystallization speed $\alpha_c(\approx K_c)$ with $\varepsilon_{c,th.}$ and C, as below:

$$K_c \approx \alpha_c = \frac{\varepsilon_{c,th.}}{C} = 0.327.$$

For the wrinkled film in the experiment, the average value of the K parameter is $0.868 > K_c$, and the film is also expected to be wrinkled according to the theoretical calculation. This result indicates that our model is roughly self-consistent.

As a starting research on the strain evolution in the perovskite films, our model is still with several simplifying assumptions and cannot be very quantitatively accurate. However, it is

still valuable to predict the trend of the strain evolution and explain the phenomena in experiments.

Supplementary note 2

Discussion on the mechanism for the surface adsorption to influence on the crystallite strain and the definition of "excessive adsorption"

There are two possible theories for the generation of the residual strain in the film. In the first theory, the residual strain originates from the mismatch between the layers solidified at different moments [13]. In a “downward” process, when the surface layer is completely solidified, there should be a certain amount of solution between the solidified surface layer and the substrate. At this moment, due to this in-between solution, there is no strain in the solidified surface layer, which is constrained by the substrate at its edge [14]. Subsequently, the volume shrinkage of the in-between solution during its solidification caused by a phase transition can result in a tensile strain in this later solidified layer but a compressive strain in the surface layer along the in-plane direction [13]. Obviously, this distribution of the residual strain is inconsistent with the ex-situ GIWAXS results in Fig. 2, and the first theory is not applicable to this work.

In the second theory, the residual strain is a result of the excessive absorption of the precursor free atoms by the solidified surface layer [14]. In the solidification process, the formation of the solidified surface film causes a chemical potential gradient in the precursor solution, leading to the diffusion of the free solute atoms to the solidified surface layer. In the polycrystalline thin film, the grain boundary is the absorption site, since the grain

boundary usually has a lower chemical potential compare to the rest of the grain surface [15].

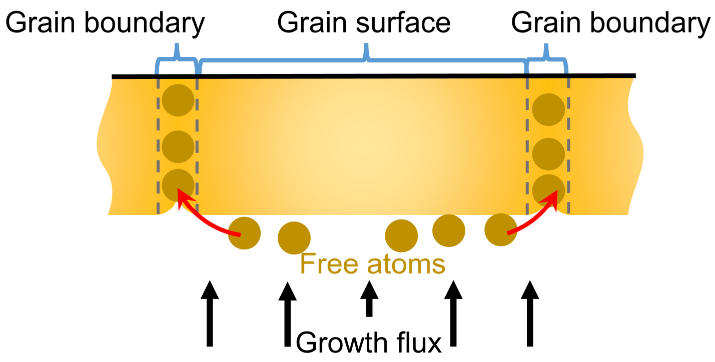

Figure S11. Schematic of model for flow of atoms into the grain boundary during film growth.

The absorption of the free atoms by the grain boundary could cause three kinds of strain states in the polycrystalline thin films during the film formation: tensile strain, no strain, and compressive strain. If the free atoms absorbed by the grain boundary just fill the boundary, there would be no strain originating from the grain boundary, which is a critical state. If more (or less) free atoms are absorbed than these in the critical state, the strain on the grain boundary would be compressive (or tensile). The excessive free atoms refer to the atoms that cause the compressive strain on the grain boundary. At the beginning, a grain boundary forms from two isolated grain, due to the new-formed grain boundary interface could reduce the total energy. Therefore, the two grains tend to coalesce, and the grain boundary is in a tensile strain due to the pulling of the two grains. As the polycrystalline thin film continuously grows, the accumulated free atom absorbed by the grain boundary

can decrease the magnitude of the tensile strain. When the absorption of the free atom reaches a critical point, there is no strain on the grain boundary. However, at this moment, the grain surface region is at a higher chemical potential than that of the grain boundary, which may result from the supersaturation of adatoms on the grain surface, nucleation of new clusters, or increased surface step density [15]. The chemical potential difference between the grain surface and the grain boundary will drive the free atom flow into the grain boundary region during the subsequent growth of the film. These additional atoms are the excessive free atoms, resulting in a compressive stress on the strain boundary. By continuously absorbing the excessive free atoms from the precursor solution, the solidified surface layer gradually expands along the in-plane direction. During the solidification process, the film will be constrained by the substrate at its edge[14], and the compressive strain along the in-plane direction will be retained as the residual strain after the completion of the solidification. Therefore, the residual compressive strain in the OIHP polycrystalline thin film originates and gradually rises with the solidification of the entire film. Because the film surface is less constrained, the residual compression strain along the in-plane direction gradually increases from the surface to the interior, which is highly consistent with the ex-situ GIWAXS results in Fig. 2. Hence, the second theory is quite applicable to the OIHP polycrystalline thin film in this work.

References

[1] W. Xing, Q. Yao, W. Zhu, H. Jiang, X. Zhang, Y. Ji, J. Shao, W. Xiong, B. Wang, B. Zhang, X. Luo, Y.Zheng, *Small* **(2021)**, *17*, e2102733.

[2] H, S. Yun, H. W. Kwon, M. J. Paik, S. Hong, J. Kim, E. Noh, J. Park, Y Lee, S. I. Seok *Nat Energy* **2020**, *7*, 828–834.

[3] A. D. Taylor, Q. Sun, K. P. Goetz, Q. An, T. Schramm, Y. Hofstetter, M. Litterst, F. Paulus, Y. Vaynzof, *Nature Communications* **2021**, *12*, 1878.

[4] J. Xing, C. Zhao, Y. Zou, W. Kong, Z. Yu, Y. Shan, Q. Dong, D. Zhou, W. Yu, C. Gu, *Light Science & Applications* **2022**, *9*, 111.

[5] Y. Cui, S. Wang, L. Ding, F. Hao, *Advanced Energy & Sustainability Research* **2021**, *2*, 2000047.

[6] D. Gedamu, I. M. Asuo, D. Benetti, M. Basti, I. Ka, S. G. Cloutier, F. Rosei, R. Nechache, *Scientific Reports* **2018**, *8*, 12885

[7] Y. Yang, H. Lu, S. Feng, L. Yang, H. Dong, J. Wang, C. Tian, L. Li, H. Lu, J. Jeong, S. M. Zakeeruddin, Y. Liu, M. Gratzel, A. Hagfeldt, E*nergy & Environmental Science* **2021**, *14*, 3447

[8] R. Guo, D. Han, W. Chen, L. Dai, K. Ji, Q. Xiong, S Li, L. K. Reb, M. A. Scheel, S. Pratap, N. Li, S. Yin, T. Xiao, S. Liang, A. L. Oechsle, C. L. Weindl, M. Schwartzkopf, H. Ebert, P. Gao, K. Wang, M. Yuan, N. C. Greenham, S. D. Stranks, S. V. Roth, R. H. Friend,P. Müller-Buschbaum, *nature energy* **2021**, *6*, 977-986

[9] S. Im, R. Huang, *Journal of the Mechanics and Physics of Solids* **(2008)**, *56*, 3315-3330.

[10] S. Wang, J. Xiao, J. Song, H. C. Ko, K.-C. Hwang, Y. Huang, J. A. Rogers, *Soft Matter* **(2010)**, *6*.

[11] D.-Y. Khang, H. Jiang, Y. Huang, J. A. Rogers, *Science* **(2006)**, *311*, 208-212.

[12] Z. Xue, H. Song, J. A. Rogers, Y. Zhang, Y. Huang, *Advanced Materials* **(2020)**, *32*, e1902254.

[13] J. A. Payne, L. F. Francis, A. V. Mccormick, *Journal of Applied Polymer Science* **1997**, *66*, 1267–1277.

[14] S. K. Basu, L. E. Scriven, L. F. Francis, A. V. McCormick, *Progress in Organic Coatings* **2005**, *53*, 1-16.

[15] E. Chason, B. W. Sheldon, L. B. Freund, J. A. Floro, S. J. Hearne, *Physical Review Letters* **2002**, *88*, 156103.
